# Chloralamid in Surgery

**Published:** 1891-09

**Authors:** Emory Lanphear

**Affiliations:** Professor of Orthopædic Surgery in the University Medical College


### Chloralamid in Surgery.

BY EMORY LANPHEAR, M.A., M.D.

Professor of Orthopædic Surgery in the University Medical College.

Extract from a clinical lecture communicated for Notes on New Remedies by the author.

Frequently after an operation of magnitude it is necessary to give the patient something to quiet the nervous system and to produce sleep. It is not always pain which causes restlessness and sleeplessness after the operation—in the majority of cases I am sure that the impression upon the nervous system, and particularly upon the mind, is what leads to the insomnia; for under our antiseptic methods, and especially where the wound has been covered with iodoform—a drug having decided anæsthetic properties—there is but a trifling amount of pain, often none, even after the most severe operative procedures. But as night draws near there is a growing restlessness, and at the hour when sleep should come the patient is anxious, nervous and wakeful. What can be done? The almost universal rule among surgeons is to order a hypodermatic injection of morphine, but I believe this is unjustifiable unless there be some indication for the anodyne effect of the opiate. This is markedly true in abdominal surgery, but in any cases the morphine is objectionable because it is apt to produce vomiting, is certain to seriously interfere with the process of digestion, is sure to induce constipation, and nearly always to give rise to headache, malaise, etc. Chloral has been suggested as a proper hypnotic; but chloral depresses the heart to a dangerous degree, and therefore can not be used in these cases. Bromides, with hyoscyamus, will sometimes answer the purpose admirably, but most stomachs rebel against this combination; so that it is hardly safe to try it. What then can we use? If a drug can be found which will be free from all these objectionable features it unquestionably will fill an important place in our materia medica.

Such a one, it seems, has been discovered in chloralamid. This comparatively new medical agent is prepared by combination of two parts of chloral hydrate with one of formamide; it is found in commerce as a colorless, crystalline substance, nearly tasteless, soluble in about twenty parts of water and two of alcohol. It will keep indefinitely in solution without decomposition, but can not be dissolved in hot solutions because of chemical changes. It acts very much like chloral and sulphonal, but does not depress the heart like the former, and is much superior to the latter in that it is soluble, exerts no bad influence upon digestion, possesses no diuretic action, never causes pruritis, vertigo, diarrhœa, or other bad symptoms which sometimes follow the administration of sulphonal—in fact, experience is demonstrating the accuracy of Rhichmann's observation: from chloralamid no ill effects in the circulation or in the feelings of patients are to be noted; and, besides, the cost is much less than that of sulphonal. T. Lauder Brunton, in a recent report on the Relative Utility of Different Hypnotics, highly recommends it and states that with reference to certainty of action and the question of tolerance chloralamid surpasses.

It exerts its influence upon both the brain and spinal cord, producing sleep and reducing the motor excitement; it may be regarded as a pure hypnotic without anodyne properties, though some late reports would indicate that it has to some degree the power for partial abolition of pain. It is, then, the ideal sedative, giving prompt and satisfactory action, reliable results and absolute freedom from evil side, or after effect.

Its dose is from fifteen to sixty grains. The proper method of exhibition is to give fifeen to thirty grains (according to the condition of the subject), repeating the dose in an hour if the first has not produced sleep; usually from ten to thirty grains give five to eight hours' refreshing slumber. The best method of giving it is to dissolve the required amount in about a teaspoonful of whisky or brandy, or in a small glass of wine if the patient prefer. It may also be given in any thing containing alcohol in considerable quantities, as tincture cardamom compound, tincture of hyoscyamus, etc. If for any reason it can not be given in this manner it may be taken in powder form, and washed down with cold water or cold tea. The direction of W. Hale White, of London, is a good one, viz.; tell the patient to dissolve the powder in brandy, add water to his liking, and drink it shortly before going to bed; this combination with spirits is particularly good in our surgical cases where whisky is usually indicated, at least in most major operations. If in any case it be better to have the medicine in liquid form, this combination may be prescribed:

R. Chloralamid . ..........................ji drachms.
Spts. frumenti...........................j fluid-ounce.
Misce bene ut ft. solut. at adde;
Syrupum rubi idæi.....................j fluid-once.

Misce. Sig. Dose one tablespoonful, to be repeated in one hour if sleep is not produced. This makes a decidedly pleasant mixture of slightly acid taste and fruity aroma and flavor.

---

Biological Action of Iron and Manganese.—Dr. Fausto Faggioli has published in a recent issue of *La Riforma Medica*, some notes of a research upon the physiological behavior of iron which he has carried out, with Professor Pellacani's assistance, in the Bologna laboratory of Forensic Medicine. From these it would appear that iron enjoys the property of setting up mitosis, or nuclear change and cellular increase, especially in cultures of unicellular organisms such as protococci. Under such circumstances manganese will act in the same way, but Dr. Faggioli was unable to find that any other metal could do so.

